# Analysis of Phosphodiesterase-5 (PDE5) Inhibitors in Modulating Inflammatory Markers in Humans: A Systematic Review and Meta-Analysis

**DOI:** 10.3390/ijms26157155

**Published:** 2025-07-24

**Authors:** Cassandra Cianciarulo, Trang H. Nguyen, Anita Zacharias, Nick Standen, Joseph Tucci, Helen Irving

**Affiliations:** 1 Holsworth Biomedical Research Centre, Department of Rural Clinical Sciences, La Trobe Rural Health School, La Trobe University, Bendigo, VIC 3550, Australia; c.cianciarulo@latrobe.edu.au (C.C.); hongtrang.nguyen@latrobe.edu.au (T.H.N.); a.zacharias@latrobe.edu.au (A.Z.); n.standen@latrobe.edu.au (N.S.); j.tucci@latrobe.edu.au (J.T.); 2 La Trobe Institute for Molecular Sciences, La Trobe University, Bendigo, VIC 3550, Australia; 3 Violet Vines Marshman Centre for Rural Health Research, La Trobe University, Bendigo, VIC 3550, Australia

**Keywords:** phosphodiesterase 5 inhibitor (PDE5), inflammation, inflammatory cytokines

## Abstract

Phosphodiesterase type 5 (PDE5) inhibitors, including sildenafil, tadalafil, and vardenafil, are primarily prescribed for erectile dysfunction and pulmonary hypertension. Emerging evidence suggests they may also modulate inflammatory pathways and improve vascular function, but their effects on inflammatory biomarkers in humans remain incompletely defined. A systematic review and meta-analysis were conducted to evaluate the impact of PDE5 inhibitors on inflammatory and endothelial markers in adult humans. Randomized controlled trials comparing PDE5 inhibition to placebo were identified through electronic database searches. Outcomes included pro-inflammatory markers (TNF-α, IL-6, IL-8, CRP, VCAM-1, ICAM-1, P-selectin) and anti-inflammatory or signalling markers (IL-10, NO, cGMP), assessed at short-term (≤1 week), intermediate-term (4–6 weeks), or long-term (≥12 weeks) follow-up. Risk of bias was assessed using the Cochrane RoB 2 tool. A total of 20 studies comprising 1549 participants were included. Meta-analyses showed no significant short-term effects of PDE5 inhibition on TNF-α, IL-6, or CRP. Long-term treatment was associated with reduced IL-6 (SMD = −0.64, *p* = 0.002) and P-selectin (SMD = −0.57, *p* = 0.02), and increased cGMP (SMD = 0.87, *p* = 0.0003). Effects on IL-10 and nitric oxide were inconsistent across studies. Most trials had low risk of bias. PDE5 inhibitors may exert anti-inflammatory effects in long-term use by reducing vascular inflammation and enhancing cGMP signalling. These findings support further investigation of PDE5 in chronic inflammatory conditions.

## 1. Introduction

Chronic inflammation is a hallmark of many pathologies, including atherosclerosis, diabetes, chronic kidney disease, and neurodegenerative diseases [1,2]. As of 2021, over 1.3 million Australians have a diagnosis of type 2 diabetes mellitus, chronic kidney disease contributes to 11% of all deaths, and dementia affects more than 400,000 people, with projections estimating over 849,000 cases by 2058 [3,4]. Persistent inflammatory states are often characterised by elevated levels of pro-inflammatory cytokines such as C-reactive protein (CRP), tumour necrosis factor-alpha (TNF-α), and interleukins (IL-6, IL-8), which contribute to disease progression and morbidity [5]. For example, IL-6 promotes hepatic CRP production and vascular cell adhesion molecule (VCAM) expression [6]; TNF-α contributes to endothelial dysfunction and insulin resistance; and IL-8 facilitates leukocyte recruitment to sites of injury [7]. Collectively, these effects contribute to a state of chronic, low-grade inflammation which underlies many age-related diseases [2,8].

Chronic inflammation plays a crucial role in the pathogenesis of endothelial dysfunction, insulin resistance, and plaque formation, all of which increase the risk of cardiovascular events [2,9]. Current strategies to manage inflammation include non-steroidal anti-inflammatory drugs, glucocorticoids, biologics (e.g., anti-IL-6 or anti-TNF therapies) and small immunomodulators (e.g., JAK inhibitors), and lifestyle-based interventions [10]. While these options may reduce inflammatory activity, many are associated with adverse effects, high costs, or limited long-term efficacy in low-grade inflammation [11,12]. Thus, alternative therapies capable of modulating inflammation more selectively and with fewer systemic side effects remain a significant area of clinical interest, and interventions that target inflammatory pathways are being investigated for their therapeutic promise. Recent studies suggest that phosphodiesterase type 5 (PDE5) inhibitors may influence these pathways, offering potential benefits beyond their vasodilatory effects [13].

PDE5 inhibitors including sildenafil (Viagra^®^), tadalafil (Cialis^®^), vardenafil (Levitra^®^), and avanafil (Stendra^®^) are commonly prescribed for erectile dysfunction and pulmonary arterial hypertension, where they act by blocking the degradation of cyclic guanosine monophosphate (cGMP), leading to increases in nitric oxide (NO) mediated signalling, causing vasodilation and smooth muscle relaxation [14]. Although primarily developed for vascular disorders, recent studies suggest that PDE5 inhibitors may also exert anti-inflammatory effects by modulating intracellular signalling pathways involved in cytokine expression [1]. In addition, these medications have garnered attention for their potential to modulate diverse inflammatory pathways, making them of interest for a broader range of clinical conditions associated with chronic inflammation, such as cardiovascular diseases, metabolic disorders, and autoimmune diseases [15].

The focus of this review is on these off-target anti-inflammatory effects. The anti-inflammatory properties of PDE5 inhibitors may be attributed to their effects on several molecular signalling mechanisms. One of these mechanisms is a primary pathway that involves the modulation of cGMP and its downstream effects on protein kinase G, which is a critical mediator of anti-inflammatory responses in various cell types, including endothelial cells and immune cells [16]. Inhibition of PDE5 has been shown to attenuate the activity of pro-inflammatory transcription factors, such as nuclear factor-kappa B, which plays a central role in the regulation of inflammatory gene expression [17]. Small increases in cellular cGMP are also associated with reductions in cytokine production by immune cell models [18]. Additionally, PDE5 inhibitors have been found to improve endothelial function and reduce oxidative stress, both of which are implicated in the inflammatory process [19,20]. By modulating these pathways, PDE5 inhibitors could potentially reduce the levels of inflammatory biomarkers, leading to clinical benefits in inflammatory diseases.

Preclinical studies have demonstrated that PDE5 inhibitors can reduce pro-inflammatory cytokine secretion and oxidative stress across a range of models, including vascular injury, metabolic disease, and organ fibrosis [21,22]. For instance, tadalafil has been shown to decrease IL-6 and TNF-α expression in rodent models of metabolic syndrome and pulmonary inflammation [21]. In a study of male salt-sensitive rats with chronic kidney disease, tadalafil reduced circulating levels of inflammatory markers, improving both endothelial function and kidney function [23]. These effects are thought to be mediated not only by nuclear factor-kappa B suppression, but also by improved endothelial function, reduced leukocyte adhesion, and enhanced NO signalling.

However, translation of these findings into human populations has been variable, and the effects of PDE5 inhibitors on systemic inflammation in clinical settings remain incompletely characterised. Several reports have suggested that PDE5 inhibitor treatment leads to significant reductions in markers of T helper cell inflammation, such as chemokine 10 in individuals with diabetic cardiomyopathy [24,25]. For instance, in patients with coronary artery disease, sildenafil was associated with a decrease in CRP and pro-inflammatory cytokines, suggesting a beneficial role in mitigating vascular inflammation [26].

In contrast, other studies have found contradictory results, showing no significant change in inflammatory biomarkers after PDE5 inhibitor treatment [27]. The reasons for these discrepancies are unclear, but may include differences in patient populations, study designs, dosages, or underlying disease mechanisms, significantly influencing the observed effectiveness of PDE5 inhibitors [27]. Individuals with elevated baseline inflammation, such as those with metabolic syndrome or cardiovascular disease, are more likely to exhibit reductions in inflammatory biomarkers, whereas low-risk or healthy participants may show minimal change [2]. Additionally, variability in treatment duration, dosing, and outcome measurement timing across studies further complicates comparisons and may contribute to inconsistent results [2]. Further, some research suggests that the duration of treatment and the presence of comorbid conditions, such as hypertension or diabetes, may influence the efficacy of PDE5 inhibitors in modulating inflammation [13].

Given the growing interest in the potential of PDE5 inhibitors to modulate inflammatory processes and their possible applications in treating conditions characterised by chronic inflammation, there is a need to critically evaluate the available evidence. Therefore, the aim of this systematic review is to consolidate and quantify the effects of PDE5 inhibitors on key inflammatory biomarkers, including pro-inflammatory markers CRP, TNF-α, IL-6, IL-8, ICAM, VCAM, and P-selectin and anti-inflammatory markers IL-10, cGMP and NO, in adult human populations. To our knowledge, this is the first systematic review and meta-analysis to comprehensively evaluate the anti-inflammatory effects of PDE5 inhibitors exclusively in human clinical trials, whereas prior reviews have largely focused on in vivo preclinical models or non-inflammatory endpoints. Therefore, this review will help improve the understanding of the impact of PDE5 inhibitors on inflammation and their potential for managing chronic inflammatory diseases.

## 2. Methods

This systematic review and meta-analysis was conducted in accordance with the guidelines outlined in the Preferred Reporting Items for Systematic Reviews and Meta-Analyses (PRISMA). The completed PRISMA checklist is provided in Supplementary S1. The protocol for this study was registered in the International Prospective Register of Systematic Reviews (PROSPERO, CDR42022384506).

### 2.1. Search Strategy and Identification of Studies

Seven databases (Embase, Cochrane, Scopus, Web of Science, PubMed, ScienceDirect, Medline) were searched systematically from the earliest date available until 17 April 2025. The search keywords were applied using three key concepts: Inflammation, Phosphodiesterase 5 inhibitor, and Immunity.

Titles and abstracts were assembled into an Excel file by one author (C.C.), and duplicate articles were excluded. Titles and abstracts were then screened independently by two authors (C.C. and H.I./A.Z./J.T./N.S.) using inclusion and exclusion criteria (Table 1). The full texts of the studies were then screened independently by three authors (C.C., H.I., and A.Z.) using the same criteria. The reviewers discussed differences in opinion until consensus was reached. Reference checking and citation tracking were also performed.

### 2.2. Population

The included studies were restricted to investigating the efficacy of phosphodiesterase 5 inhibitors on pro- and anti-inflammatory outcomes in adult individuals over 18 years of age and healthy or with chronic health conditions.

### 2.3. Intervention

Included studies were those that administered a PDE5 inhibitor (e.g., sildenafil citrate, tadalafil, or vardenafil) or a visually identical placebo, delivered in tablet or infusion form, to adult participants through randomised allocation.

### 2.4. Comparisons

Included studies were required to compare a control group receiving placebo with an intervention group receiving a PDE5 inhibitor, with no other active comparators permitted (Table 2).

### 2.5. Outcomes

Outcomes included measures of inflammatory cytokine (TNF-α, IL-6, IL-8), anti-inflammatory cytokine (IL-10), cGMP production, NO production, high-sensitivity CRP (hs-CRP) production, intercellular adhesion molecule (ICAM), VCAM, and P-selectin.

### 2.6. Research Design

Study types included randomized clinical trials and prospective observational studies that reported in peer-reviewed journals.

### 2.7. Data Extraction

Data including study design, participants, description of intervention, intervention duration, and outcome measure from each included study were extracted into a Excel spreadsheet. Data extraction was performed by one author (C.C.) and verified by two authors independently (T.N. and H.I.). In cases where there were no numerical data provided in the studies, the data were qualitatively assessed from graphs using visual scales and averages. If there were no data in the studies, data were requested from the original author.

### 2.8. Quality Analysis

The updated Cochrane Risk of Bias tool (RoB 2.0) was used to rate the methodological quality of all included studies. Three judgment items were considered: low, unclear/some concerns, and high risk of bias, across five RoB domains (bias arising from the randomisation process, any deviations from the intended interventions, missing outcome data, bias in measurement of outcomes, and selection of reported results) (Supplementary Table S4). Risk of Bias analysis was performed by one author (C.C.) and verified by a second author (T.N.).

### 2.9. Data Synthesis

In order to answer questions about PDE5 inhibitor efficiency through pro- and anti-inflammatory outcomes, studies were grouped for meta-analysis based on duration of intervention in the ST, IT, or LT regardless of patient population and therapeutic dose (Table 2). Data collected were converted into mean ± standard deviation as described in Wan et al. [28] if not provided in that format. The relevant data were extracted and manually entered into Review Manager (RevMan, web version 5, https://revman.cochrane.org (accessed on 11 June 2025) by one author (C.C.) and checked by a second author (T.N.) for meta-analysis. RevMan 5.3 software was used to perform the meta-analyses. Where appropriate, heterogeneity was assessed, and subgroup analyses were performed to explore potential sources of variation. Standardised mean difference (SMD) and 95% confidence intervals (CIs) were calculated for outcome measures of interest to indicate the effect size. The I^2^ statistic was used for assessing heterogeneity. A value of 0% was interpreted as indicating no observed heterogeneity, and a value of 100% was considered a completely heterogeneous sample. Values of 25%, 50%, and 75% indicated low, moderate, and high levels of heterogeneity. In cases where meta-analysis was not possible due to limited data or incompatible reporting formats, results were synthesized narratively.

## 3. Results

### 3.1. Yield

**Figure 1:**
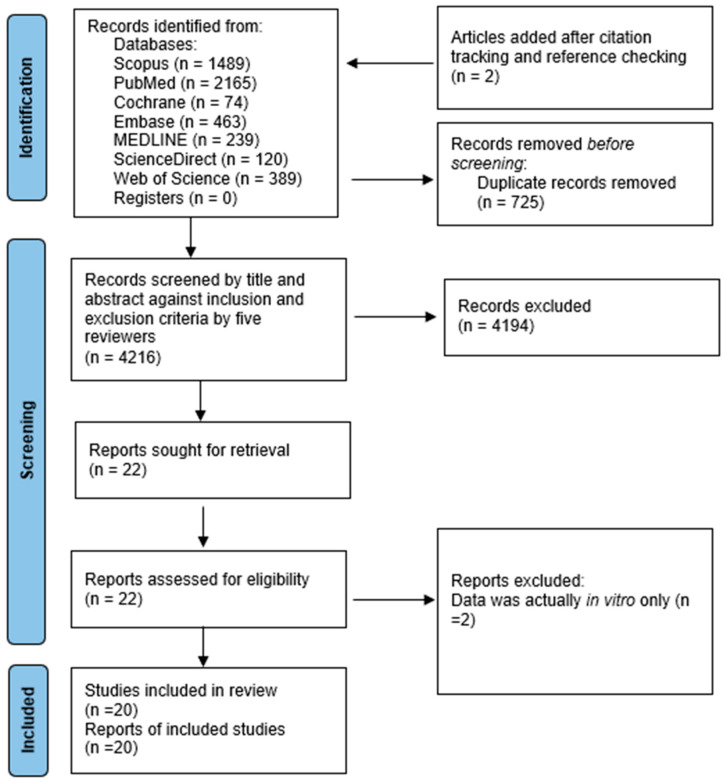
PRISMA flowchart indicating the progress of search study selection.

A total of 4214 studies were identified through database searches; 725 duplicate studies that appeared in more than one database were removed. Citation tracking and reference checking added an additional two studies. The studies underwent screening of titles and abstracts based on study selection criteria, and as a result, 22 studies were selected (Figure 1). After full-text screening, 20 studies [13,26,29,30,31,32,33,34,35,36,37,38,39,40,41,42,43,44,45,46] were included in the final review (Supplementary Table S2). Studies excluded from full-text screening and the reasons for exclusion can be found in Supplementary Table S3. Fourteen of the 20 full text-articles were included for meta-analysis, with the remaining six narratively synthesised in text.

### 3.2. Characteristics of Included Studies

The studies included in this systematic review encompass randomized controlled trials, placebo-controlled interventions, and prospective observational designs evaluating the effects of PDE5 inhibitors on inflammatory, metabolic, and vascular parameters in adult humans. Study populations were heterogeneous and included individuals with type 2 diabetes mellitus, erectile dysfunction, diabetic cardiomyopathy, chronic obstructive pulmonary disease, pulmonary artery hypertension, cystic fibrosis, and benign prostatic hyperplasia, as well as overweight but otherwise healthy individuals. Intervention durations ranged from acute single-dose administration to chronic regimens extending up to 24 weeks, with PDE5 inhibitors including sildenafil, tadalafil, and vardenafil at doses ranging from 10 mg to 100 mg daily.

Inflammatory markers assessed across studies included hs-CRP, interleukins (IL-6, IL-8), TNF-α, and cell adhesion molecules ICAM-1, VCAM-1, and P-selectin. In addition, several included studies measured levels of anti-inflammatory markers (IL-10) and signalling molecules (cGMP, NO) (Supplementary Table S1).

Risk of bias was accessed across all 20 included studies using the Cochrane Risk of Bias 2 (ROB 2) tool (Supplementary Table S4). The majority of studies were rated as having low risk of bias across key domains, including randomisation, deviations from intended interventions, missing outcome data, measurement of the outcome, and selection of reported results. Random sequence generation and allocation concealment were clearly described in most trials. Blinding of participants, personnel, and outcome assessors was implemented in most placebo-controlled trials.

### 3.3. Effect of PDE5 Inhibitors on Inflammatory Markers

#### 3.3.1. Short-Term Effects of PDE5 Inhibitors on Pro-Inflammatory Outcomes

**Figure 2:**
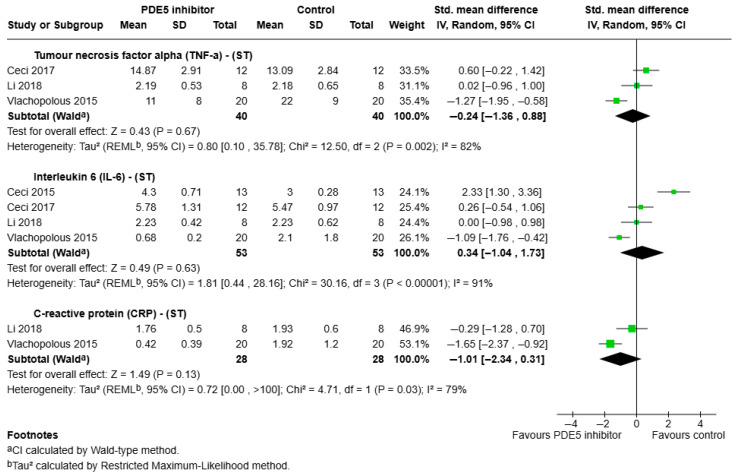
Forest plot illustrating the effects of short-term PDE5 inhibitor administration (<1 week) on pro-inflammatory biomarkers including IL-6, CRP, and TNF-α. Standardised mean differences (SMD) with 95% confidence intervals are shown [26,31,32,40].

a. TNF-α:

Three studies reported short-term (<1 week) effects on TNF-α outcomes (Figure 2). Meta-analysis showed no significant difference in TNF-α levels between PDE5 inhibitor and placebo groups (SMD = −0.24, 95% CI: −1.36 to 0.88, *p* = 0.67), with high heterogeneity (I^2^ = 82%).

b. IL-6:

IL-6 was reported in four studies (Figure 2), with no significant effect observed for PDE5 inhibitor treatment in the short term (SMD = 0.34, 95% CI: −1.04 to 1.73, *p* = 0.63) when compared to placebo treatment. Heterogeneity was high (I^2^ = 91%). A single study [39] that could not be included in the meta-analysis also reported no significant changes in IL-6 concentrations following intravenous PDE5 administration during cardiac surgery.

c. IL-8:

Due to substantial differences in study design and intervention delivery, a meta-analysis could not be performed for short-term IL-8 outcomes. Two single studies reported IL-8 responses following PDE5 inhibitor administration. In one trial, plasma IL-8 levels were significantly reduced 6 h after tadalafil treatment compared to both baseline (*p* < 0.05) and placebo at the same timepoint (*p* < 0.01) [31]. In contrast, another study reported no significant change in IL-8 concentrations following intravenous sildenafil infusion during surgery [39].

d. CRP:

Meta-analysis (two studies, Figure 2) showed a non-significant reduction in CRP following PDE5 inhibitor administration (SMD = −1.01, 95% CI: −2.34 to 0.31, *p* = 0.13), with high heterogeneity (I^2^ = 79%).

e. VCAM:

Only a single study [26] examined VCAM-1 levels and found no significant difference between sildenafil and placebo at 2 or 4 h post-administration in men with vasculogenic erectile dysfunction.

#### 3.3.2. Intermediate—Long Term Effects of PDE5 Inhibitors on Pro-Inflammatory Outcomes

**Figure 3:**
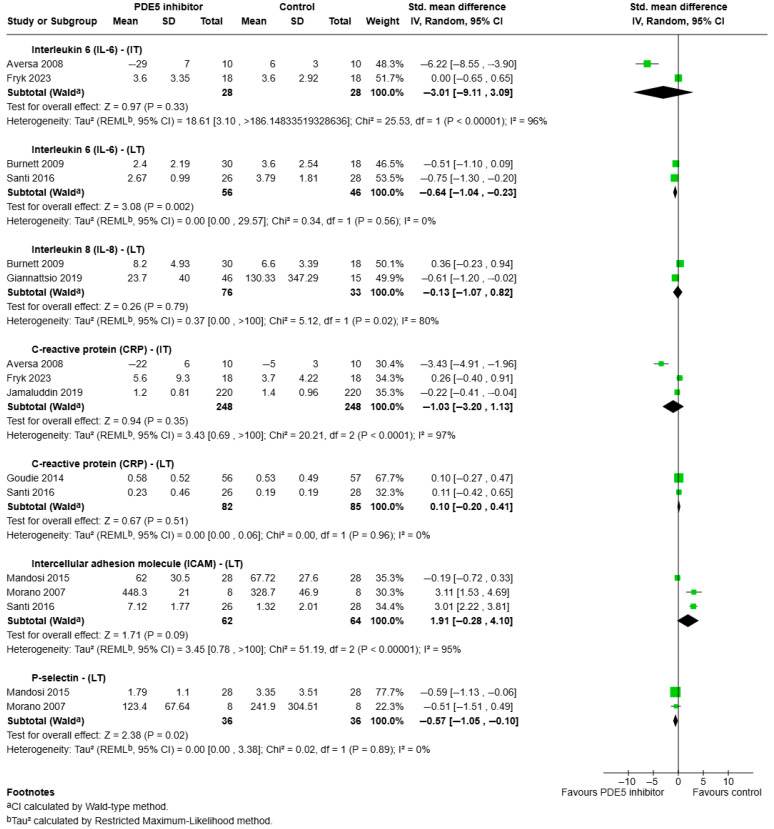
Forest plot depicting the effects of intermediate (≥1 week to 6 weeks) to long-term (>12 weeks and up to 24 weeks) PDE5 inhibitor treatment on systemic inflammatory biomarkers. The analysis includes IL-6, IL-8, CRP, ICAM, and P-selectin. Standardised mean differences (SMD) with 95% confidence intervals are shown [29,30,34,35,36,37,41,42,44].

a. IL-6:

The effects of PDE5 on IL-6 were assessed in two studies at intermediate-term and two studies at long-term (Figure 3). No significant effect was observed at intermediate-term (SMD = −3.01, 95% CI: −9.11 to 3.09, *p* = 0.33; I^2^ = 96%). However, long-term PDE5 inhibitor treatment was associated with a significant reduction in IL-6 levels, with no heterogeneity (SMD = −0.64, 95% CI: −1.04 to −0.23, *p* = 0.002; I^2^ = 0%).

b. IL-8:

IL-8 was measured in two long-term studies (Figure 3), which showed no significant change in levels following PDE5 inhibitor administration (SMD = −0.13, 95% CI: −1.07 to 0.82, *p* = 0.79), with high heterogeneity (I^2^ = 80%).

c. CRP:

CRP was reported in three intermediate-term studies and two long-term studies (Figure 3). Meta-analysis of studies that measure CRP at intermediate-term showed no significant difference in CRP, with high heterogeneity (SMD = −1.03, 95% CI: −3.20 to 1.13, *p* = 0.35; I^2^ = 97%), and long-term analysis similarly showed no significant difference, with minimal heterogeneity (SMD = −0.10, 95% CI: −0.20 to 0.41, *p* = 0.52; I^2^ = 0%), following PDE5 inhibitor treatment.

d. ICAM:

Meta-analysis of three studies (Figure 3) that measured ICAM-1 at long-term following PDE5 inhibitor treatment showed no significant difference between groups, with high heterogeneity (SMD = 1.91, 95% CI: −0.28 to 4.10, *p* = 0.09; I^2^ = 95%). Additionally, a single intermediate-term study by Aversa et al. [29] observed significant reductions in ICAM-1 levels following 4 weeks of PDE5 inhibitor therapy in men with type 2 diabetes.

e. P-selectin:

P-selectin was assessed in two long-term studies (Figure 3). Meta-analysis indicated a significant reduction in P-selectin levels following PDE5 inhibitor treatment (SMD = −0.57, 95% CI: −1.05 to −0.10, *p* = 0.02), with low heterogeneity (I^2^ = 0%).

f. TNF-α:

TNF-α was reported in a single intermediate-term study [34]. No significant change in TNF-α levels was detected after 6 weeks of high-dose 20 mg per day tadalafil in patients with type 2 diabetes.

g. VCAM:

VCAM-1 was measured in two single studies at intermediate-term and long-term. Aversa et al. [29] reported significant reductions in VCAM-1 after 4 weeks (intermediate-term) of sildenafil therapy in diabetic men compared to placebo, while Santi et al. [44] found no significant difference in VCAM-1 levels after 24 weeks (long-term) of vardenafil treatment in a similar clinical population.

#### 3.3.3. Effects of PDE5 Inhibitors on Anti-Inflammatory Outcomes

**Figure 4:**
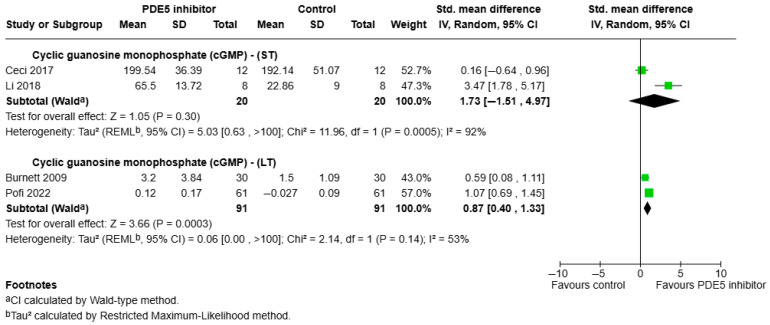
Forest plot showing the effects of PDE5 inhibitor administration on cGMP outcomes across short-term and long-term treatment periods. Standardised mean differences (SMD) with 95% confidence intervals are shown [30,31,40,43].

a. cGMP:

cGMP concentrations were reported in four studies (Figure 4). The pooled analysis of these short-term studies showed no significant difference in cGMP levels in the PDE5 inhibitor group compared to placebo (SMD = 1.73, 95% CI: −1.51 to 4.97, *p* = 0.30), with substantial heterogeneity (I^2^ = 92%). In contrast, two long-term studies showed a significant elevation in cGMP levels with PDE5 inhibitor treatment (SMD = 0.87, 95% CI: 0.40 to 1.33, *p* = 0.0003), with moderate heterogeneity (I^2^ = 53%).

b. IL-10:

Due to differences in clinical context and drug administration, a meta-analysis could not be performed for this outcome. IL-10 was measured in two single studies. No statistically significant differences in IL-10 plasma concentrations were observed at 2, 6, or 24 h following a single 20 mg dose of tadalafil in healthy adult men [31]. Similarly, postoperative IL-10 concentrations remained unchanged in patients who received intravenous sildenafil during cardiac surgery [39].

c. NO:

Due to heterogeneity in treatment duration, administration of PDE5 inhibitor, and participant populations, a meta-analysis could not be performed for NO outcomes. NO and its metabolites (nitrate, nitrite) were assessed across three studies investigating PDE5 inhibitor effects. No significant changes in NO outcomes were observed following short-term tadalafil administration in healthy men or after intravenous sildenafil use during surgery [31,39]. In contrast, chronic sildenafil treatment in men with type 2 diabetes resulted in a significant increase in plasma nitrate/nitrite levels (*p* < 0.05) [29].

#### 3.3.4. Other Inflammatory Outcomes

Several studies could not be included in the meta-analysis due to insufficient data being reported or the use of surrogate inflammatory endpoints. Corinaldesi et al. [33] explored the effects of long-term daily tadalafil administration in a cohort of patients with metabolic hypogonadism and mild systemic inflammation. In this study, reductions in inflammatory markers such as C-X-C motif chemokine 10 (CXCL10) were reported following 16 weeks of treatment, alongside improvements in testosterone levels. Kilic et al. [38] examined systemic inflammation in the context of erectile dysfunction treatment responsiveness, using haematological ratios including monocyte-to-high-density lipoprotein cholesterol ratio and lymphocyte-to-monocyte ratio as biomarkers. Although not all patients demonstrated significant changes in inflammatory profiles, those who responded positively to PDE5 inhibitor treatment generally presented with lower baseline inflammatory status. In a study by Semen et al. [45], sildenafil treatment over 12 weeks in patients with pulmonary arterial hypertension resulted in marked reductions in oxidative stress markers, including hydroxynonenal and altered fatty acid composition. Taylor-Cousar et al. [46] conducted a six-week open-label study of sildenafil in adults with cystic fibrosis and mild to moderate lung disease. The most notable immunological finding was a significant reduction in sputum elastase activity, a marker of neutrophilic inflammation. Lastly, Vignozzi et al. [13] investigated the anti-inflammatory effects of PDE5 inhibition in men with lower urinary tract symptoms secondary to benign prostatic hyperplasia. In vivo, both treatments significantly reduced stromal infiltration of CD45+ leukocytes, an established marker of overall inflammatory cell burden within the prostate tissue. This reduction indicates a suppression of leukocyte recruitment and immune activation in the hyperplastic prostate environment, suggesting that PDE5 inhibition may exert local anti-inflammatory effects in addition to its known urodynamic benefits. Stratification by metabolic status revealed that men with metabolic syndrome had significantly higher CD45 scores than those without (*p* = 0.002), indicating greater baseline inflammatory burden.

## 4. Discussion

This systematic review and meta-analysis provides new insights into the anti-inflammatory potential of PDE5 inhibitors in humans, demonstrating a selective, time-dependent suppression of inflammatory mediators, most notably IL-6 and IL-8. The strongest effects were observed in intermediate- and long-term studies involving individuals with cardiometabolic comorbidities [35,44], while short-term interventions and healthy populations yielded less consistent changes [31,32]. This pattern suggests that both cumulative drug exposure and baseline inflammatory activity are crucial to the therapeutic responsiveness of inflammatory biomarkers.

IL-6 and IL-8 reductions in long-term studies likely reflect the central role of these cytokines in chronic low-grade inflammation, particularly in metabolic syndrome, endothelial dysfunction, and insulin resistance [5]. Mechanistically, PDE5 inhibition enhances intracellular cGMP levels, which activates protein kinase G and subsequently inhibits nuclear factor-kappa B translocation, a key transcriptional regulator of IL-6 and IL-8 gene expression [15]. This mechanism has been confirmed in vitro where tadalafil and vardenafil were shown to reduce IL-8 secretion in prostate tissue via a protein kinase G-dependent pathway [13]. In clinical settings, similar reductions in systemic IL-8 were demonstrated in patients with diabetic cardiomyopathy [35].

By contrast, short-term studies, including both single-dose [39] and multi-dose interventions [26,31,32,40], did not consistently demonstrate reductions in IL-6, suggesting that a longer duration of exposure may be necessary to modulate cytokine transcription and immune cell recruitment. This was observed where no significant changes in IL-6 or TNF-α were found within hours of sildenafil or tadalafil dosing [26,32]. These findings are aligned with the pharmacodynamics of PDE5 inhibitors, where transient cGMP elevation may enhance vasodilation but not sustain the transcriptional and redox changes required for immunomodulation [47]. Although short-term PDE5 inhibitor treatment did not significantly reduce key inflammatory biomarkers such as IL-6 or TNF-α, it remains unclear whether this reflects a true absence of anti-inflammatory activity or a temporal delay in biomarker response relative to clinical improvement. Most included studies did not report functional or clinical outcomes alongside biomarker data, limiting our ability to assess this relationship.

CRP and TNF-α, although central to inflammatory signalling, were modulated less consistently across studies [32,33,37]. TNF-α reductions were reported in some acute and chronic studies [26,33], but the lack of reproducibility may stem from its short half-life, upstream role in cytokine cascades, or inter-individual variability [48]. Although TNF-α is itself a cytokine, its binding to TNF receptors 1 and 2 initiates complex intracellular signalling cascades, including the NF-κB, c-Jun N-terminal, and p38 mitogen pathways, which in turn regulate the expression of numerous other cytokines and inflammatory mediators, amplifying its downstream effects [49]. CRP, a hepatic acute-phase protein induced by IL-6, may require longer periods to reflect systemic changes [50]. Decreased CRP levels were reported after 16 weeks of tadalafil in hypogonadal men with metabolic syndrome, while no significant changes were observed in shorter studies or those involving participants with lower baseline inflammation [33,36,37].

NO and its metabolites (nitrate/nitrite) were also not consistently affected by PDE5 inhibition [29,31,39] despite their central role in cGMP signalling and vascular homeostasis. This discordance may reflect compartmental differences between endothelial and systemic NO levels or the fact that PDE5 inhibition prevents cGMP breakdown rather than directly stimulating NO synthesis [47]. Increased nitrate/nitrite levels were reported following intracavernosal sildenafil in diabetic men, while no significant changes in NO were observed in studies using oral or intravenous PDE5 inhibitor administration [29,32,39].

The narrative synthesis also revealed tissue-specific and mechanistically diverse anti-inflammatory effects. Sildenafil was shown to reduce neutrophil elastase activity in cystic fibrosis patients, suggesting suppression of airway neutrophilia, while a 12-week course of sildenafil reduced lipid peroxidation markers and improved fatty acid profiles in patients with pulmonary hypertension [45,46]. These findings align with the hypothesis that PDE5 inhibitors can influence not only cytokine release but also broader oxidative and immune networks.

An additional layer of complexity is introduced by emerging markers such as the monocyte-to-high-density lipoprotein ratio [51]. This marker has been identified as a predictor of therapeutic response to PDE5 inhibitors in erectile dysfunction [38], reinforcing the concept that systemic inflammatory burden can modulate vascular responsiveness and possibly drug efficacy.

When synthesised with preclinical and mechanistic studies, the findings from this systematic review support the repurposing of PDE5 inhibitors as immunomodulatory agents, particularly in populations with cardiometabolic disease, systemic inflammation, or endothelial dysfunction [13,47]. However, the heterogeneity of study design, population health status, treatment duration, and biomarker selection complicates the establishment of broadly applicable effects. Furthermore, mechanistic and condition-specific trials are needed to clarify the pathways through which PDE5 inhibitors may exert anti-inflammatory effects, and to determine whether these findings translate into meaningful clinical benefit. Ultimately, these data suggest that PDE5 inhibitors interact with inflammatory signalling in a biomarker-specific, time-dependent, and disease-contextual manner. Their therapeutic potential in inflammation-driven diseases is promising but will depend on careful matching of drug pharmacokinetics to disease pathophysiology and mechanistic outcome measures.

### Strengths and Limitations

To our knowledge, this review is the first to critically examine the anti-inflammatory effects of PDE5 inhibitors across human clinical studies encompassing a wide range of treatment durations, populations, and biomarker outcomes. Furthermore, outcomes were assessed in relation to time frame and baseline disease context, which provided important insight into the temporal and population-specific dynamics of PDE5 effects on inflammation.

However, the included studies exhibited considerable heterogeneity in terms of study design, PDE5 inhibitor type and dose, treatment duration, and participant characteristics. With the exception of Kumar et al. [39], all doses of PDE5 inhibitors were within normal therapeutic dosage ranges for erectile dysfunction, ranging from 25 mg to 100 mg orally daily for sildenafil and 10 mg to 20 mg daily for tadalafil. Kumar et al. [39] used a single intravenous 12.5 mg dose of sildenafil, which is estimated to be equivalent to 30 mg orally. A potential limitation would be the duration of action of each PDE5 inhibitor, ranging from 6 to 8 h for sildenafil and up to 36 h for tadalafil [52,53], meaning patients exposed to tadalafil would be more likely to show sustained effects. Many trials evaluated inflammation as a secondary outcome, in part to provide biological context, mechanistic validation, or exploratory data regarding the systemic effects of PDE5 inhibitors in diseases where inflammation is a known contributor to pathogenesis. To mitigate heterogeneity in future studies, standardisation of biomarker selection, treatment duration, and dosing regimens is essential. Consistent timing of outcome assessments and the use of validated assay methods would improve comparability across trials. Clearer inclusion criteria, along with stratified analyses based on baseline inflammation, comorbidities, and population type may help account for clinical variability. Additionally, improved reporting of numerical data, rather than graphical summaries alone, would enhance the accuracy of data synthesis in future reviews. These issues limited our ability to perform subgroup analyses and likely contributed to the high between-study variance observed in some meta-analyses. Although we stratified findings by time frame to partially account for these differences, few studies reported repeated measures or longitudinal biomarker trajectories, restricting our understanding of temporal response patterns.

Several potentially relevant studies were excluded from quantitative synthesis due to non-standardised outcome reporting or insufficient data, and although these were incorporated narratively, their exclusion from meta-analysis may have introduced selection bias. Moreover, outcome measures such as nitric oxide were often assessed using indirect surrogates (e.g., nitrate/nitrite), which may not reflect biologically active or compartment-specific NO levels and therefore may underestimate or misrepresent the true extent of PDE5 inhibitor-induced changes in NO signalling, particularly within endothelial or tissue-specific microenvironments.

Publication bias could not be formally assessed due to the small number of studies per outcome and may have influenced the overall direction of findings. Finally, while the review aimed to explore inflammatory effects in humans, there was some variation in the primary indication for PDE5 inhibitor treatment (e.g., erectile dysfunction, pulmonary hypertension, cystic fibrosis, benign prostatic hyperplasia), which introduces potential confounding factors, as the pathophysiology, inflammatory pathways, and clinical contexts differ across these conditions, making appropriate interpretation harder.

## 5. Conclusions

This systematic review and meta-analysis provides evidence that PDE5 inhibitors exert selective anti-inflammatory effects in humans, particularly through the downregulation of IL-6 and IL-8. These effects were most consistently observed in intermediate- and long-term treatment durations and in clinical populations with underlying metabolic or cardiovascular inflammation. By contrast, inflammatory outcomes in short-term studies or healthy individuals were often negligible, reinforcing the role of disease context and sustained pharmacologic exposure in modulating immune responses.

Mechanistically, these findings align with preclinical data indicating that PDE5 inhibition enhances cGMP signalling, which can suppress nuclear factor-kappa B activity and reduce pro-inflammatory cytokine production. Moreover, several studies demonstrated improvements in markers of oxidative stress, endothelial function, and neutrophil activity, supporting broader immunomodulatory effects beyond cytokine regulation.

However, outcomes such as CRP, TNF-α, and NO were more variably affected, likely reflecting differences in biomarker half-life, tissue compartmentalisation, and sensitivity to drug-induced changes. Additionally, exploratory markers such as the monocyte-to-high-density lipoprotein ratio offer new avenues for identifying patients more likely to benefit from PDE5 inhibitor therapy.

The interpretation of these findings is complicated by heterogeneity in study designs, drug pharmacokinetics, treatment durations, populations, and primary indications. Since the inflammatory response varies substantially between conditions such as erectile dysfunction, pulmonary hypertension, and cystic fibrosis, the applicability of findings across disease contexts remains limited. Future research should prioritise mechanistically driven, stratified trials that incorporate inflammation as a primary endpoint, ideally supported by tissue-specific analyses, consistent drug and dosage regimens, and clinically meaningful outcomes.

Collectively, these results support the growing view that PDE5 inhibitors may hold therapeutic promise beyond vasodilation, with potential to be repositioned as adjunctive treatments in inflammation-driven diseases, particularly those characterised by endothelial dysfunction and chronic low-grade inflammation.

## Figures and Tables

**Table 1:** Study inclusion criteria.

Criteria
Must contain the concept of inflammation, regulation, or intervention of inflammation.
Must contain the concept of Phosphodiesterase-5 inhibitors.
Must contain primary or secondary outcome markers of inflammation.
Must be human studies.
Must be adults over 18 years of age.
Must not contain in vitro or in vivo (animal) studies.
Must not contain review articles.
Must be peer-reviewed studies.
Must be in English.

**Table 2:** Basis for comparisons by meta-analysis.

Duration of Outcome Measurement After Intervention	Comparison Groups	Outcome Measures
Short term (ST)—Less than 1 week	Pre and post intervention	TNF-α, IL-6, CRP, cGMP
Intermediate term (IT)—4–6 weeks	Pre and post intervention	IL-6, CRP
Long term (LT)—Equal to or more than 12 weeks	Pre and post intervention	IL-6, IL-8, CRP, cGMP, ICAM, P-selectin

## Data Availability

Data are contained within the article.

## Supplementary Materials

The following supporting information can be downloaded at: https://www.mdpi.com/article/10.3390/ijms26157155/s1. References [47,54,55,56,57,58,59,60,61,62,63,64] are cited in the supplementary materials.

## Abbreviations

The following abbreviations are used in this manuscript:

PDE5	Phosphodiesterase 5
TNF-α	Tumour necrosis factor alpha
IL-6	Interleukin 6
IL-8	Interleukin 8
IL-10	Interleukin 10
NO	Nitric oxide
cGMP	Cyclic guanosine monophosphate
VCAM	Vascular cell adhesion molecule
ICAM	Intracellular adhesion molecule
CRP	C-reactive protein
ST	Short term
IT	Intermediate term
LT	Long term
CI	Confidence interval
SMD	Standardised mean differences
SD	Standard deviation
