# Supplementary material for: Analysis of Phosphodiesterase-5 (PDE5) Inhibitors in Modulating Inflammatory Markers in Humans: A Systematic Review and Meta-Analysis
Source: Int J Mol Sci. 2025 Jul 24;26(15):7155. doi: 10.3390/ijms26157155 (PMC12345901; doi:10.3390/ijms26157155)
Supplement: Supplementary file 1 [file ijms-26-07155-s001.zip › Supplementary S2.pdf]

Supplementary Materials

Analysis of Phosphodiesterase-5 (PDE5) Inhibitors in Modulating Inflammatory Markers in Humans: A Systematic Review and Meta-Analysis

Cassandra Cianciarulo, Trang H. Nguyen, Anita Zacharias, Nick Standen, Joseph Tucci and Helen Irving

Index

Table S1. Description of outcome biomarkers used in included studies. p2

Table S2. Characteristics of included studies. p3

Table S3. List of excluded articles and reasons. p7

Table S4. Risk of bias of included studies. p8

Reference list p9

Supplementary Materials

Table S1. Description of outcome biomarkers used in included studies.

Abbreviation	Name	Effect	Immune effect
PDE5	Phosphodiesterase 5 inhibitor	Block the action of the enzyme PDE5. [1]	N/A
CRP	C-reactive protein	Produced in the liver from stimulation from IL-6, binds to cells to activate complement system. [2]	Pro-inflammatory
TNF-α	Tumour necrosis factor alpha	Produced by macrophages (primarily), induces inflammation via TNF receptors. [3]	Pro-inflammatory
IL-6	Interleukin 6	Secreted by macrophages in response to specific microbial molecules, referred to as pathogen-associated molecular patterns (PAMPs). [4]	Pro-inflammatory
IL-8	Interleukin 8	Chemokine involved in attracting neutrophils to sites of inflammation and other immune responses. [5]	Pro-inflammatory
ICAM	Intercellular adhesion molecule	Cell surface glycoprotein involved in cell adhesion and immune responses, particularly during inflammation. [6]	Pro-inflammatory
VCAM	Vascular cell adhesion protein	Mediates the adhesion of lymphocytes, monocytes, eosinophils and basophils to vascular endothelium. [7]	Pro-inflammatory
P-Selectin	P-selectin	Cell adhesion molecule belonging to the selectin family, expressed on activated platelets and endothelial cells. [8]	Pro-inflammatory
IL-10	Interleukin 10	Produced by various immune cells, such as macrophages and lymphocytes. Helps to prevent excessive inflammation and autoimmunity. [9]	Anti-inflammatory
NO	Nitric oxide	Produced by nitric oxide synthases, activates cGMP production causing vasodilation. Free radical which causes DNA damage in targeted cells. Reduces immune cell function. [10]	Anti-inflammatory
cGMP	Cyclic Guanosine Monophosphate	Intracellular secondary messenger, upon activation by NO catalyses. [11]	Anti-inflammatory

Table S2. Characteristics of included studies.

No	Study	Clinical population and sample size	Stimulant	Intervention duration	Comparison groups	Outcome measure of interest	Effect size Std. mean difference, IV, Random, 95% CI
1	Aversa *et al.* (2008)	Men with type 2 diabetes (n=20)	Sildenafil	3 days 100mg/day, 4 days 25mg/day, 3 weeks 25mg/day	Placebo & Intervention	Nitric oxide<br>IL-6<br>CRP<br>ICAM<br>VCAM	(Negative favours intervention)<br>IL-6: -6.22 (-8.55, -3.90)<br>(Negative favours intervention)<br>CRP: -3.43 (-4.91, -1.96)
2	Burnett *et al.* (2009)	Men with type 2 diabetes and erectile dysfunction (n=292)	Sildenafil	Phase 1: 1-week 50mg/day, 3 weeks 100mg/day<br>Phase 2: 2 weeks 50mg/day, 10 weeks either 25mg, 50mg or 100mg/day (self-managed)	Placebo & Intervention	cGMP<br>IL-6<br>IL-8	(Positive favours intervention)<br>cGMP: 0.59 (0.08, 1.11)<br>(Negative favours intervention)<br>IL-6: -0.51 (-1.10, 0.09)<br>(Positive favours control)<br>IL-8: 0.36 (-0.23, 0.94)
3	Ceci *et al.* (2015)	Healthy males undertaking exhaustive exercise (n=13)	Tadalafil	48 hours 20mg/day	Placebo & Intervention	IL-6	(Positive favours control)<br>IL-6: 2.33 (1.3, 3.36)
4	Ceci *et al.* (2017)	Healthy males (n=12)	Tadalafil	24 hours 20mg/day	Placebo & Intervention	NO<br>cGMP<br>IL-6<br>IL-8<br>IL-10<br>TNF-α	(Positive favours intervention)<br>cGMP: 0.16 (-0.64, 0.96)<br>(Positive favours control)<br>IL-6: 0.26 (-0.54, 1.06)<br>(Positive favours control)<br>TNF-α: 0.6 (-0.22, 1.42)
5	Corinaldesi *et al.* (2021)	Men and women with systemic sclerosis (n=116)	Sildenafil	Part of treatment regimen for serious cases	Placebo & Intervention	CXCL10	Not estimable.

6	Fryk *et al.* (2023)	Men and women with type 2 diabetes (n=36)	Tadalafil	6 weeks 20mg/day	Placebo & Intervention	CRP<br>TNF-α<br>IL-6	(Positive favours control)<br>CRP: 0.26 (-0.40, 0.91)<br>(No favour)<br>IL-6: 0.00 (-0.65, 0.65)
7	Giannattasio *et al.* (2018)	Men with diabetic cardiomyopathy (n=61)	Sildenafil	3 months 100mg/day	Placebo & Intervention	IL-8	(Negative favours intervention)<br>IL-8: -0.61 (-1.20, -0.02)
8	Goudie *et al.* (2014)	Men and women with chronic pulmonary disease (COPD) (n=120)	Tadalafil	12 weeks 10mg/day	Placebo & Intervention	CRP	(Positive favours control)<br>CRP: 0.10 (-0.27, 0.47)
9	Jamaluddin *et al.* (2019)	Men with erectile dysfunction (n=220)	Tadalafil	6 weeks 10mg/day	No placebo, Before and after intervention	CRP	(Negative favours intervention)<br>CRP: -0.22 (-0.41, -0.04)
10	Kilic *et al.* (2023)	Men with erectile dysfunction (n=185)	Either of Sildenafil or Tadalafil	4 days of either 100mg/day sildenafil or 20mg/day tadalafil	No placebo, Before and after intervention	NLR (neutrophil/lymphocyte ratio)<br>LMR (lymphocyte/monocyte ratio)<br>PLR (platelet/lymphocyte ratio)<br>MHR (monocyte/high-density lipoprotein cholesterol ratio)<br>Neutrophils<br>Lymphocytes<br>Monocytes	Not estimable.

11	Kumar *et al.* (2020)	Men and women with acute kidney injury undergoing cardiac surgery (n=125)	Sildenafil	12.5mg/150 minutes (given during surgery)	Placebo & Intervention	IL-10<br>IL-6<br>IL-8<br>NO	Not estimable.
12	Li *et al.* (2018)	Overweight men (n=16)	Sildenafil	1-week 100mg/day	Placebo & Intervention	cGMP<br>TNF-α<br>IL-6<br>CRP	(Positive favours intervention)<br>cGMP: 3.47 (1.78, 5.17)<br>(Positive favours control)<br>TNF-α: 0.02 (-0.96, 1.0)<br>(No favour)<br>IL-6: 0.00 (-0.98, 0.98)<br>(Negative favours intervention)<br>CRP: -0.29 (-1.28, 0.70)
13	Mandosi *et al.* (2015)	Men with type 2 diabetes (n=28)	Sildenafil	3 months 100mg/day	No placebo, Before and after intervention	P-selectin<br>ICAM	(Negative favours intervention)<br>P-selectin: -0.59 (-1.13, -0.06)<br>(Negative favours intervention)<br>ICAM: -0.19 (-0.72, 0.33)
14	Morano *et al.* (2007)	Men with type 2 diabetes and erectile dysfunction (n=32)	Sildenafil	12 weeks 50mg/twice weekly	Placebo & Intervention	P-selectin<br>ICAM	(Negative favours intervention)<br>P-selectin: -0.51 (-1.51, 0.49)<br>(Positive favours control)<br>ICAM: 3.11 (1.53, 4.69)
15	Pofi *et al.* (2022)	Men and women with long duration diabetes (n=122)	Tadalafil	20 weeks 20mg/day	Placebo & Intervention	cGMP	(Positive favours intervention)<br>cGMP: 1.07 (0.69, 1.45)
16	Santi *et al.* (2016)	Men with type 2 diabetes and erectile dysfunction (n=54)	Vardenafil	24 weeks 10mg/day	Placebo & Intervention	CRP<br>ICAM<br>VCAM<br>IL-6	(Positive favours control)<br>CRP: 0.11 (-0.42, 0.65)<br>(Positive favours control)<br>ICAM: 3.01 (2.22, 3.81)<br>(Negative favours intervention)<br>IL-6: -0.75 (-1.30, -0.20)

17	Semen *et al*. (2016)	Men and women with pulmonary arterial hypertension (n=14)	Sildenafil	12 weeks at 25mg/three times a day	Placebo & Intervention	4-hydroxynonenal (4HNE)	Not estimable.
18	Taylor-Cousar *et al*. (2015)	Men and women with mild to moderate cystic fibrosis lung disease (n=20)	Sildenafil	6 weeks of either 20mg/day or 40mg/day	No placebo, Before and after intervention	Sputum elastase activity	Not estimable.
19	Vignozzi *et al*. (2013)	Men with benign prostatic hyperplasia ± metabolic syndrome (n=43)	Vardenafil	12 weeks 10mg/day	Placebo & Intervention	Anti-pan leukocytes (CD45)	Not estimable.
20	Vlachopoulos *et al*. (2015)	Men with erectile dysfunction (n=20)	Sildenafil	Over either 2, 4 or 8 hours 100mg	Placebo & Intervention	CRP<br>IL-6<br>VCAM-1<br>TNF-α	(Negative favours intervention)<br>CRP: -1.65 (-2.37, -0.92)<br>(Negative favours intervention)<br>IL-6: -1.09 (-1.76, -0.42)<br>(Negative favours intervention)<br>TNF-α: -1.27 (-1.95, -0.58)

Table S3. List of excluded articles and reasons.

**Reasons for exclusion**	**Articles**
In vitro data only	1. Kohzadi, R.; Cheraghi, E.; Mehranjani, M.S.; Shariatzadeh, M. Sildenafil citrate ameliorates the adverse effects of cryopreservation on sperm quality in asthenozoospermic men. Cryobiology 2023, 111, 126-133, doi:10.1016/j.cryobiol.2023.05.005.<br>2. Di Luigi, L.; Sgrò, P.; Duranti, G.; Sabatini, S.; Caporossi, D.; Del Galdo, F.; Dimauro, I.; Antinozzi, C. Sildenafil Reduces Expression and Release of IL-6 and IL-8 Induced by Reactive Oxygen Species in Systemic Sclerosis Fibroblasts. Int J Mol Sci 2020, 21, doi:10.3390/ijms21093161.

Table S4. Risk of bias of included studies.

Key: 1= Risk of bias arising from the randomization process, 2= Risk of bias due to deviations from the intended interventions (effect of assignment to intervention, 3= Missing outcome data, 4= Risk of bias in measurement of the outcome, 5= Risk of bias in the selection of the reported result. L= Low, H= High, SC= Some concerns.

**Articles**	**Domain 1**	**Domain 2**	**Domain 3**	**Domain 4**	**Domain 5**	**Overall Risk of bias**
Aversa *et al.* (2008)	L	L	L	L	L	L
Burnett *et al.* (2009)	L	L	L	L	L	L
Ceci *et al.* (2015)	L	L	L	L	L	L
Ceci *et al.* (2017)	L	L	L	L	L	L
Corinaldesi *et al.* (2021)	L	L	SC	L	L	L
Fryk *et al.* (2023)	L	L	L	L	L	L
Giannattasio *et al.* (2018)	L	L	L	L	L	L
Goudie *et al.* (2014)	L	L	L	L	L	L
Jamaluddin *et al.* (2019)	L	L	SC	L	L	L
Kilic *et al.* (2023)	L	SC	L	L	L	L
Kumar *et al.* (2020)	L	L	L	L	L	L
Li *et al.* (2018)	L	L	L	L	L	L
Mandosi *et al.* (2015)	L	L	L	L	L	L
Morano *et al.* (2007)	L	L	L	L	L	L
Pofi *et al.* (2022)	L	L	SC	L	L	L
Santi *et al.* (2016)	L	L	L	L	L	L
Semen *et al.* (2016)	H	SC	SC	SC	L	SC
Taylor-Cousar *et al.* (2015)	SC	SC	SC	L	SC	SC
Vignozzi *et al.* (2013)	L	L	L	L	L	L
Vlachopoulos *et al.* (2015)	L	L	SC	L	L	L

References

1. Ahmed, W.S.; Geethakumari, A.M.; Biswas, K.H. Phosphodiesterase 5 (PDE5): Structure-function regulation and therapeutic applications of inhibitors. *Biomedicine & Pharmacotherapy* **2021**, *134*, 111128, doi:https://doi.org/10.1016/j.biopha.2020.111128.
2. Sproston, N.R.; Ashworth, J.J. Role of C-Reactive Protein at Sites of Inflammation and Infection. *Frontiers In Immunology* **2018**, *9*, 754, doi:10.3389/fimmu.2018.00754.
3. Jang, D.I.; Lee, A.H.; Shin, H.Y.; Song, H.R.; Park, J.H.; Kang, T.B.; Lee, S.R.; Yang, S.H. The Role of Tumor Necrosis Factor Alpha (TNF-α) in Autoimmune Disease and Current TNF-α Inhibitors in Therapeutics. *International Journal of Molecular Sciences* **2021**, *22*, doi:10.3390/ijms22052719.
4. Hirano, T. IL-6 in inflammation, autoimmunity and cancer. *International Immunology* **2020**, *33*, 127-148, doi:10.1093/intimm/dxaa078.
5. Matsushima, K.; Yang, D.; Oppenheim, J.J. Interleukin-8: An evolving chemokine. *Cytokine* **2022**, *153*, 155828, doi:https://doi.org/10.1016/j.cyto.2022.155828.
6. Bui, T.M.; Wiesolek, H.L.; Sumagin, R. ICAM-1: A master regulator of cellular responses in inflammation, injury resolution, and tumorigenesis. *Journal of Leukocyte Biology* **2020**, *108*, 787-799, doi:10.1002/jlb.2mr0220-549r.
7. Kong, D.H.; Kim, Y.K.; Kim, M.R.; Jang, J.H.; Lee, S. Emerging Roles of Vascular Cell Adhesion Molecule-1 (VCAM-1) in Immunological Disorders and Cancer. *International Journal of Molecular Sciences* **2018**, *19*, doi:10.3390/ijms19041057.
8. Ye, Z.; Zhong, L.; Zhu, S.; Wang, Y.; Zheng, J.; Wang, S.; Zhang, J.; Huang, R. The P-selectin and PSGL-1 axis accelerates atherosclerosis via activation of dendritic cells by the TLR4 signaling pathway. *Cell Death & Disease* **2019**, *10*, 507, doi:10.1038/s41419-019-1736-5.
9. Iyer, S.S.; Cheng, G. Role of interleukin 10 transcriptional regulation in inflammation and autoimmune disease. *Critical Reviews In Immunology* **2012**, *32*, 23-63, doi:10.1615/critrevimmunol.v32.i1.30.
10. Kumar, S.; Singh, R.K.; Bhardwaj, T.R. Therapeutic role of nitric oxide as emerging molecule. *Biomedicine & Pharmacotherapy* **2017**, *85*, 182-201, doi:https://doi.org/10.1016/j.biopha.2016.11.125.
11. Francis, S.H.; Busch, J.L.; Corbin, J.D. cGMP-Dependent Protein Kinases and cGMP Phosphodiesterases in Nitric Oxide and cGMP Action. *Pharmacological Reviews* **2010**, *62*, 525-563, doi:https://doi.org/10.1124/pr.110.002907.
